# The Analysis of FBG Central Wavelength Variation with Crack Propagation Based on a Self-Adaptive Multi-Peak Detection Algorithm

**DOI:** 10.3390/s19051056

**Published:** 2019-03-01

**Authors:** Weifang Zhang, Meng Zhang, Xiangyu Wang, Yan Zhao, Bo Jin, Wei Dai

**Affiliations:** 1 School of Reliability and Systems Engineering, Beihang University, 37 Xueyuan Rd., Haidian Dist., Beijing 100191, China; 08590@buaa.edu.cn (W.Z.); zhangmeng123@buaa.edu.cn (M.Z.); 2 School of Energy and Power Engineering, Beihang University, 37 Xueyuan Rd., Haidian Dist., Beijing 100191, China; wangxiangyu2016@buaa.edu.cn (X.W.); zy_buaa@buaa.edu.cn (Y.Z.); 3 Information Science Academy of China Electronics Technology Group Corporation, Building 4, No. 36, North Rd., Sidaokou, Haidian Dist., Beijing 100086, China; by1504121@buaa.edu.cn

**Keywords:** FBG sensor, central wavelength, peak detection algorithm, structural health monitoring

## Abstract

We propose a peak seeking algorithm to extract the damage characteristic-variation of central wavelength to monitor the crack damage status in aluminum alloy plates using surface bonded fiber Bragg grating (FBG) sensors. The FBG sensors are sensitive to the uniform and non-uniform strain distribution along their longitudinal direction, and the effect appears in the power spectrum of the reflected light from the gauge section. In this paper, we propose a fast-self-adaptive multi-peak seeking algorithm to detect the central wavelength shifting of the FBG reflection spectrum with the crack propagation. The proposed peak searching algorithm results point to a significant improvement compared to other conventional methods. Then the central wavelength shifting is applied to explain the crack propagation behavior of the aluminum plates under quasi-static tensile test conditions. The different damages feature changing intervals which are associated with the crack position and the FBGs location, demonstrating that central wavelength shifting performs as an indicator to detect structural crack damage.

## 1. Introduction

Structure health monitoring (SHM) is a critical process for enhancing structural integrity or validity. Sensing technologies using various sensors for intelligent structure have also been investigated [1]. FBG sensors have shown broad application prospects in structure health monitoring due to their advantages, such as small size, light weight, high resolution, multiplexing capability and immunity to electromagnetic fields [2]. Furthermore, FBG sensors have shown great potential for monitoring strain [3], vibration [4] and temperature [5], thus various studies based on the FBG sensitivity characteristics have been performed to detect different environmental states as strain sensors, temperature sensors, etc. Recently, FBG sensors have attracted more attention for crack detection in composite [6] and metallic materials [7]. In particular, the stress concentrated sections caused by crack damages in structures must be monitored. During a crack event, different fracture features will be present near the crack tip, such as a stress gradient and a tensile field [8]. Then, grating gauges can sense inhomogeneous stress/strain and the reflection spectrum of the bonded FBG sensor, which includes information on strain distribution, which is useful for monitoring the damage status. Therefore, this study mainly seeks to link the crack damage status directly to the damage features extracted from the reflection spectrum. In order to focus on the unique spectrum change variables that affect the stress/strain changes caused by the crack damage, then the effect of the other factors, such as the planar Bragg gratings [9], the cross-effect between temperature and strain [10] and curvature effects [11] have been taken into consideration by using healthy spectra which were acquired in a no crack state as the reference signals. Then the deformation spectrum can be compared with the basic signal to eliminate the impact of other factors.

The proposed approach has two key advantages over previous studies. First, the proposed self-adaptive multi-peak algorithm can effectively extract the damage features of central wavelength shifts and the experimental results show that the performance of the proposed algorithm is superior to that of other traditional peak seeking algorithms in pm accuracy and self-adaption. Second, the different characteristic variation intervals can be associated with different crack propagation states by a detailed analysis. Additionally, the damage feature proposed in this paper represents the average strain loaded in the gratings and has a connection with the crack length.

The traditional peak seeking algorithms such as the maximum and extremum algorithm [12], first-order derivative [13] and the thresholding methods [14] have many limitations, such as poor anti-noise performance, low computational accuracy. The peak searching methods based on curve fitting, including Gaussian fitting [15], polynomial fitting [16], three-point peak detection [17] and the centroid algorithm [18] have high peak detection precision, but their performances are affected by spectral types, especially for deformation asymmetry spectra. To overcome the mentioned problems, other algorithms were developed, however, for the super-Gaussian model [19] it is difficult to choose the modified function parameters and the Monte Carlo methods [20] can hardly meet the precision demands owing to their nonlinear characteristics. Additionally, several optimization algorithms such as genetic algorithm [21], self-adaptive neighborhoods search [19], tree search [22], and dynamic multi-swarm particle optimizer algorithm [23] have been proposed to deal with the multi-peak detection problem. However, their iterations take a long time to find the optimum solution and their computational complexity is high. Therefore, it is desirable to develop a multi-peak seeking method to extract the central wavelength shifts from the FBG reflection spectrum. 

The proposed algorithm can be adapted to extract the damage characteristic, the central wavelength shift which is used to detect the hole-crack damage under fatigue loading. The holes drilled in plates for assembly or functional requirements will cause regional stress concentrations and lead to further varying degrees of structural damage under external loading [24]. For the purpose of monitoring such a complicated damage state, many studies have experimentally and by simulation examined the reflection spectrum of embedded FBG sensors during damage extension in composite laminates, which contain local stress singularity regions [25,26]. Then, the damage characteristics such as central wavelength [27], full width at half maximum (FWHM) [28] and the peak numbers [29] are reported to detect different damages include debonding [30], ply cracks [31] and delaminations [32]. However, few studies have focused on the linkage of the transverse fatigue hole-edge crack growth in aluminum alloys plates and the associated FBG sensing characteristics. This paper attempts to show the strain gradient which is formed along the grating length when the crack tip approaches it, and produces a variation in the grating pitch. Then the central wavelength damage feature which corresponds to the located direct-peak will drift with the crack propagation [21]. Finally, a detailed analysis of the relation between the crack length and the central wavelength shift enables us to monitor damage status at any loading condition. 

The paper is organized as follows: in Section 2, a fast-self-adaptive peak seeking algorithm is proposed to detect the central wavelength by four processes. In Section 3, we present the experimental design of the fatigue crack monitoring based on FBG sensors. In Section 4, the performances of different peak seeking algorithms are evaluated and the detected feature variations with crack propagation are discussed in details. Finally, Section 5 concludes the paper.

## 2. Central Wavelength Detection Algorithm

External loads can lead to uniform or non-uniform strain field distributions along the sensor grating. Takeda [33] found that the central wavelength of FBG reflection spectrum shifts are proportional to the applied strain (assuming isothermal conditions) when the strain field is uniform, however, when the grating is under a non-uniform strain, the reflection spectrum becomes broadened and shows multiple peaks with the shifting central wavelength. Dong [34] proposed that the damage features extracted from the healthy and deformation spectrum be used to detect, locate and predict the structure crack evolution. Chong [35] monitored multi-crack locations based on a FBG sensing network and the relationship between the central wavelength and the compression strain along fiber gratings simultaneously. The traditional peak seeking algorithms mentioned before have various disadvantages, such as sensitivity to experimental noise and spectrum shape, low computation power and being time consuming. Therefore, it necessary to propose a fast and precise damage feature detection algorithm to monitor the central wavelength shifting and analyse its variation with the expansion of cracks.

**Figure 1:**
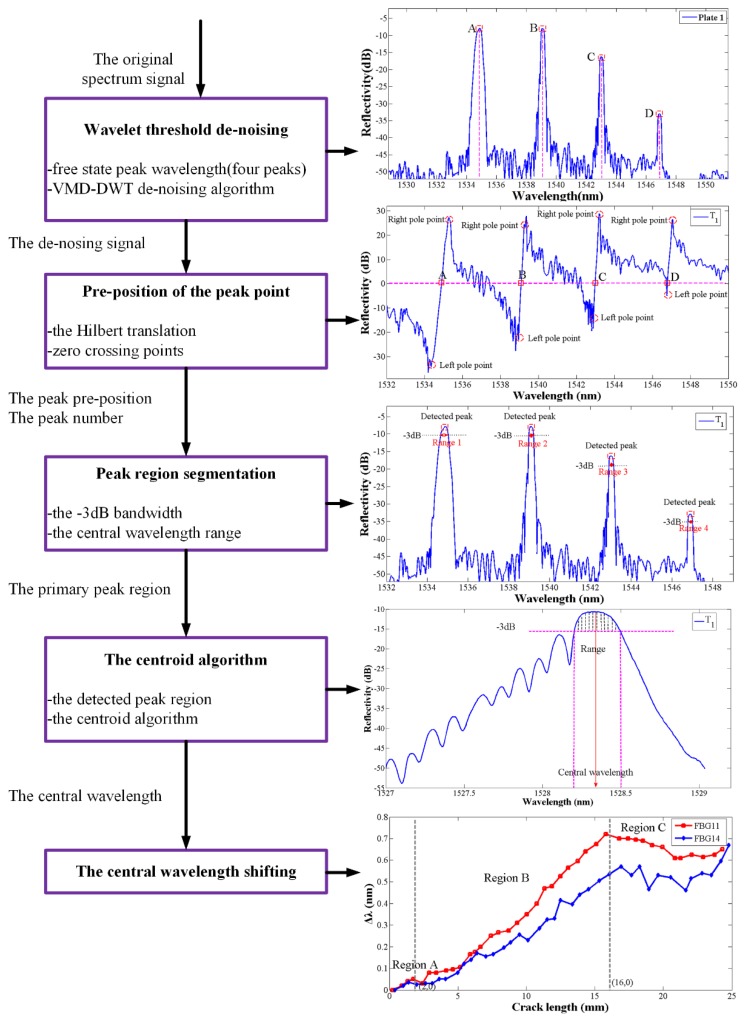
Flow chart of the primary peak detection algorithm.

In this paper, a fast-central wavelength seeking algorithm is proposed. First, the improved wavelet thresholding combined with variational mode decomposition method is applied to denoise the FBG signals. Then, the peak range detection method is proposed to confirm the number and scope of target peaks in a multi-peak spectrum. When the crack propagation is near the FBG sensors, the non-homogeneous strain field results in spectrum broadening and a shift in the spectrum peak. Considering the subordinate peaks and the low powered clutter, it is necessary to quantity the range of the primary peak at the 3 dB bandwidth region. Finally, the central wavelength is obtained by the centroid algorithm and the shifting is defined based on a comparison between the healthy and damage spectra. The kernel of the algorithm presented in this paper is the peak-recognition which is developed by fully considering the shape characteristics of spectra, demonstrated to be effective as a deformation spectrum. The flowchart of the central wavelength shifting detection algorithm is shown in Figure 1.

### 2.1. De-Noising Pre-treatment of the FBG Reflectivity Spectrum

The FBG sensing system is subject to interferences from external noise caused by electrical devices and the loading environment that affects the peak shape. The peak features such as spectral broadening, asymmetry, top fluctuation and side lobes then limit the peak detection precision. It is suggested that smooth de-noising should effectively eliminate top fluctuation. Therefore, FBG signal de-noising before peak detection is necessary. A variational mode decomposition combined with the wavelet threshold denoising method is proposed in this paper to deal with the FBG signals. In 2014, Konstantin [36] first introduced an entirely non-recursive variational mode decomposition (VMD), which can concurrently extract the modes which are related to the center frequencies $w_{k}$ on the line from the input signal. The basic modes are called band-limited intrinsic mode functions (BLIMFs) $u_{k}$, corresponding to the sub-energy of the signal. When the decomposition number is predefined, the energy distribution in each basic mode is decided. Then, inspired by the translation invariant wavelet widely used in signal processing technology, the VMD combined with changed wavelet thresholding denoising techniques is developed in this paper. 

Based on the previous research [37], the process of the VMD-DWT signal de-noising algorithm is described as follows:

Step 1: The best decomposition number *K* is set as 6 and the best balance parameter α is equal to 200. An input signal with length *N* is decomposed into the given number *K* band-limited intrinsic mode functions (BLIMFs) $u_{k}$ by VMD.

Step 2: A translate wavelet is executed using thresholding for each BLIMF. Based on literature research, the high-order BLIMFs parts which are significantly corrupted by noise can be locally excluded. Then, the DWT system 5 is adopted as the wavelet basic, and the decomposition level is set as 6 [38]. According to the previous research, soft thresholding shows greater efficacy than the hard one in FBG signals [38]. Thus, in this paper, soft thresholding is applied to all BLIMF samples. Additionally, when the samples’ extrema exceed the threshold, the extrema need to get reduced by an amount equal to the threshold in a smooth way. When considering the energy distribution impact in each basic mode $u_{k}$, the improved wavelet thresholding is expressed in the Equation (1):

(1) $$\hat{d_{l}}=\{\begin{array}{l} sign\left(d_{i}\right)\left(\left|d_{i}\right|-\frac{T_{i}}{\exp{\left(\frac{\left|d_{i}\right|}{T}-1\right)}^{2}}\right),\;\left|d_{i}\right|\geq T_{i}\;\\ 0,\;\left|d_{i}\right|<T_{i} \end{array}$$

where, $d_{i}$ presents the $i_{th}$ thresholding of BLIMFs, and the standard deviation of the noise is estimated by the components median $\sigma =\frac{median\left(\left|c_{i}\right|:i=1,2\ldots .N\right)}{0.675}$. Additionally, the per mode thresholding $T_{i}$ which is corresponding to the variance noise energy are described in Equation (2):

(2) $$T=100\sigma \sqrt{2E_{i}\mathrm{lg}\left(N\right)}/\mathrm{lg}\left(j+1\right)$$

Here, the $E_{i}$ shows the $i_{th}$ energy of BLIMFs and it can be estimated by the following equation:

(3) $${\hat{E}}_{k}=\frac{E_{1}^{2}}{\beta }\rho^{-k},\;(k=2,3,4)$$

Here, $E_{1}^{2}$ is the energy of the first BLIMFs and the shifting iterative procedure parameters β,ρ are specifically given as 0.719 and 2.01 [39].

Step 3: After the processes of de-noise on each scale separately, then, we reconstruct the de-noising signal by summing all the threshold denoising BLIMFs. 

**Figure 2:**
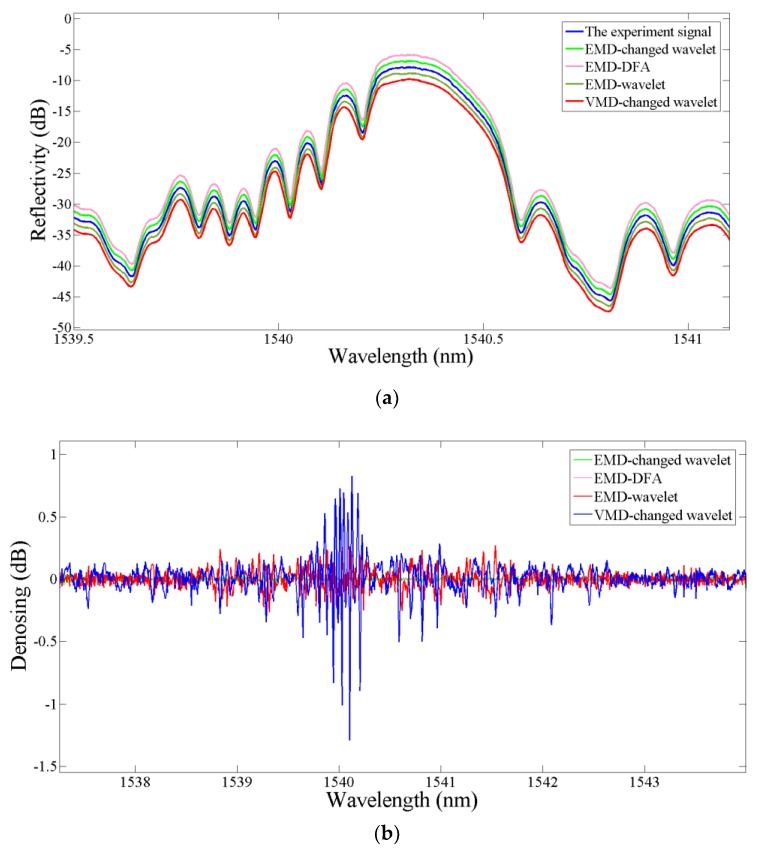
(**a**). FBG reflection spectrum with noise; (**b**) The de-noised spectrum signal of FBG by the proposed algorithm.

Processed by the improved wavelet method proposed in this paper, the noise contained in spectral signal is effectively removed, laying the foundation for the multi-peak detection operation. Figure 2 shows the evaluation results of the de-noising performance with different methods, such as the Empirical Mode Decomposition combined with the changed thresholding wavelet (EMD-changed wavelet), EMD combined with detrended fluctuation analysis (EMD-DFA), EMD-wavelet, VMD-changed wavelet. It is apparent that the VMD-changed wavelet method efficiently filters the noises and conserves details from the partial superposition signals. 

### 2.2. Flow of the Multi-Peak Central Wavelength Detection Algorithm

**Figure 3:**
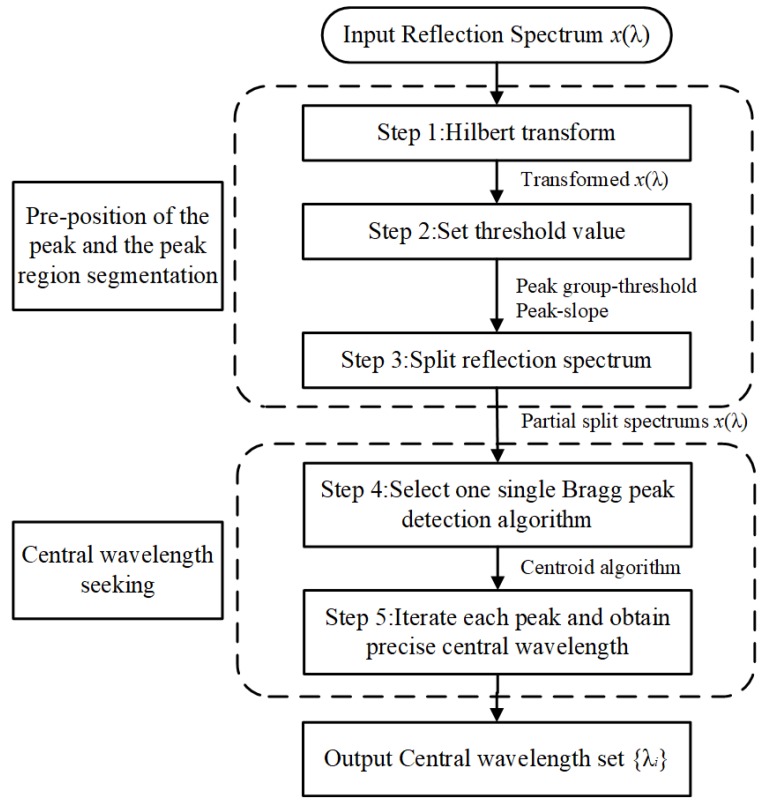
Algorithm flow of the multi-peak central wavelength detection algorithm.

The multi-peak central wavelength detection algorithm is used to handle the issue of precise multi-peak detection, particularly with regards to the deformation reflection spectrum. The improved algorithm is divided into two-sub-tasks: multi-peak splitting and central wavelength detection. The first section is a spectral splitter that splits the reflection spectrum and seeking the multi-peak pre-positions. The second section is a Bragg wavelength detector that extracts the central wavelength from each split spectrum. Those two sections contain six steps as the algorithm flow in Figure 3 shows.

#### 2.2.1. Pre-position of the Peak and the Peak Region Segmentation

In this section, the de-noised spectral signal are processed with the Hilbert transform to achieve the peak number and pre-position detection of the multi-peak spectral signal. According to the previous research [40], the peak position of the reflection spectrum corresponds to the zero-crossing points of the transformed signal. 

The Hilbert transform in the time domain is a convolution integral between the real continuous spectrum signal x(t) and ${\left(\pi t\right)}^{-1}$. Then we denote the Hilbert transform of x(t) as Equation (4):

(4) $$\hat{x}\left(t\right)=H\left[x\left(t\right)\right]=x\left(t\right)*\frac{1}{\pi t}$$

According to one of the Hilbert transform relations, the phase spectrum is only different between $\hat{x}\left(t\right)$ and x(t), when the amplitude spectrum and energy spectrum are the same.

**Figure 4:**
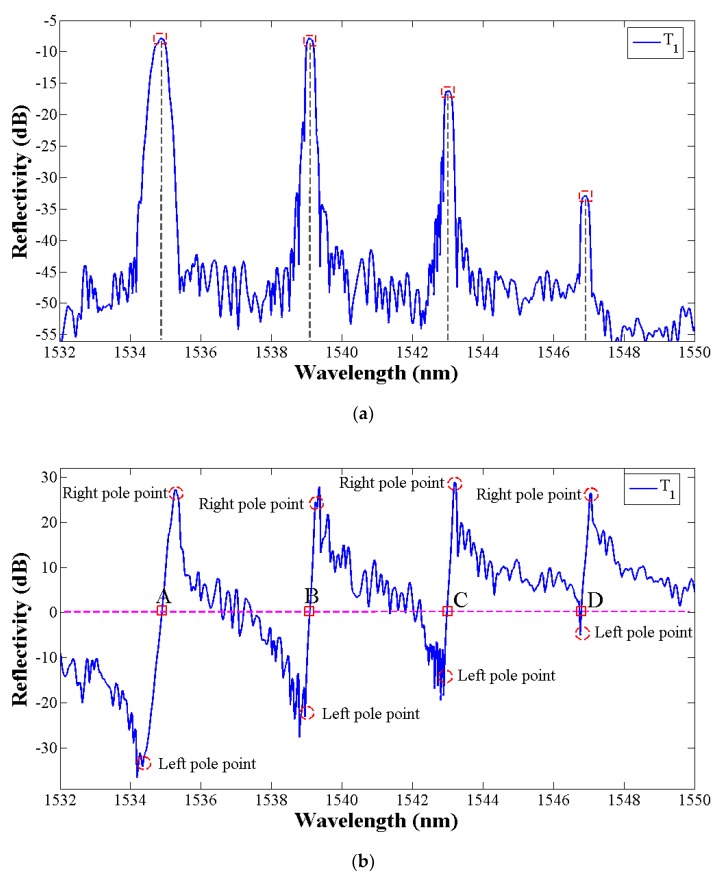
(**a**) The FBG reflection spectrum; (**b**) The spectrum signal after the Hilbert transform.

The reflection spectra of four FBGs in plate 1 are shown in Figure 4a and the Hilbert transform signal results are shown in Figure 4b. It can be seen that the central wavelength of each peak in Figure 4a corresponds to the zero-crossing points of the transformed signal in Figure 4b. However, the zero-crossing points also be related to the extreme points in FBGs reflection spectrum. Then the peak group-threshold and the peak-slope is proposed to eliminate the interference of subordinate peaks. The peak group is a vector of the number of points around the top part of the peak that are taken as the initial estimate of the peak center and width, which is defined by the boundary length of the pole points on both sides of the transformed signal in Figure 4b. According to the previous research [41], the noise can limit the peak threshold, so to avoid the noise and the sidelobe effect, O’Haver [42] proposed the autopeaksplot algorithm and definite the minimized primary peak threshold in one peak cycle range. Considering the data number collected by SM125 at one pause is 16,001, therefore in this paper, the peak threshold data number is set as 600 and the wavelength length approaches 1 nm. In the interval between the left and right pole points in Figure 4b, the intensity of the transformed signal $\hat{x}\left(\lambda \right)$ has a sharp increasing shape, which can be expressed as a large positive slope. The following peak detection through looking for zero-crossings points in the Hilbert transform signal whose peak-thresholds exceed 1 nm and in a sharply increasing slope shape. Additionally, the spectral intensity of x(λ) in Figure 4a and $\hat{x}\left(\lambda \right)$ in Figure 4b have the same fluctuation trend in the FBG reflection peak region. By this way, the peak number and the peak pre-position in the reflection spectrum can be estimated. $T_{1}$

**Figure 5:**
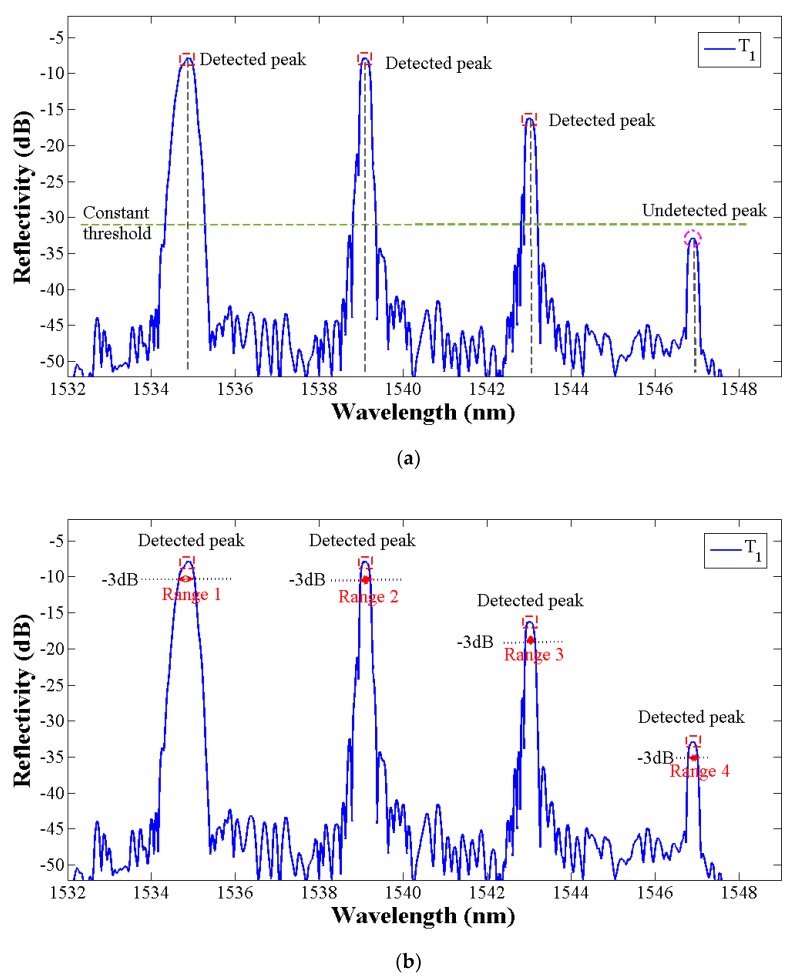
(**a**) Conventional peak detection using a global threshold. (**b**) Peak detection using proposed algorithm.

Compared with the traditional constant thresholding peak detection method, the proposed automatic peak detection algorithm can overcome the limitation that the lower value peaks are not detected and false spurious peaks resulting from the existence of sidelobes or peak distortion (shown in Figure 5a) are detected. The Hilbert transform of the FBG signals returned the peak number and the peak pre-judgment position. Then, the 3 dB threshold bandwidth which is associated with the half-power bandwidth of the peak is defined as the peak range with the purpose of precision central wavelength detection (shown in Figure 5b). 

#### 2.2.2. Central Wavelength Seeking

**Figure 6:**
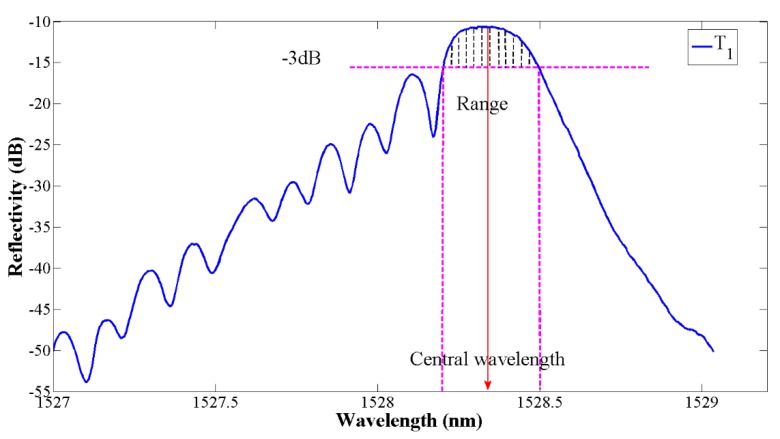
Schematic of the central wavelength detection by centroid algorithm.

Based on the pre-position of the peak and the peak region segmentation, a 3 dB window function is added to suppress the side lobes in the pre-processing. Then, the centroid algorithm is used to detect the precise central wavelength in multi-peak (in Figure 6). Compared with other algorithms, the centroid algorithm has a high precision and is more robust than the direct-peak location algorithm when noise raw data is processed [41]. Additionally, the centroid algorithm requires higher computation than the Gaussian or polynomial fitting in the FBG reflection spectrum [43]. The centroid algorithm produces a point corresponding to the geometric centroid of a spectrum, calculated by Equation (5), where *N* is the size of the spectrum points vector, $\lambda_{i}$ is the $i_{th}$ point wavelength, and $I_{i}$ is the $i_{th}$ point reflectivity intensity:

(5) $$\lambda_{B}=\frac{\sum_{i=1}^{N}\lambda_{i}I_{i}}{\sum_{i=1}^{N}I_{i}}$$

## 3. Damage Monitoring Experiment Design

The experimental platform with surface bonded FBG sensors on an aluminium alloy plate is set up to monitor the hole-edge crack states by analysing the damage characteristics which are extracted from healthy and deformation reflectivity spectra. The crack defects will reduce the light intensity and disturb light transmission. Then, the central wavelength shift $∆\lambda_{B}$ is a direct indication of the strain along the specimen. By analysing the changing damage features, it was observed that the magnitude of $∆\lambda_{B}$, measured by each FBG, increases after the crack passes the grating area, and has a relationship with the crack lengths. Additionally, the experiment results will also be used to verify the effectiveness of the proposed peak seeking algorithm. 

### 3.1. Specimens: Material and Geometry

**Figure 7:**
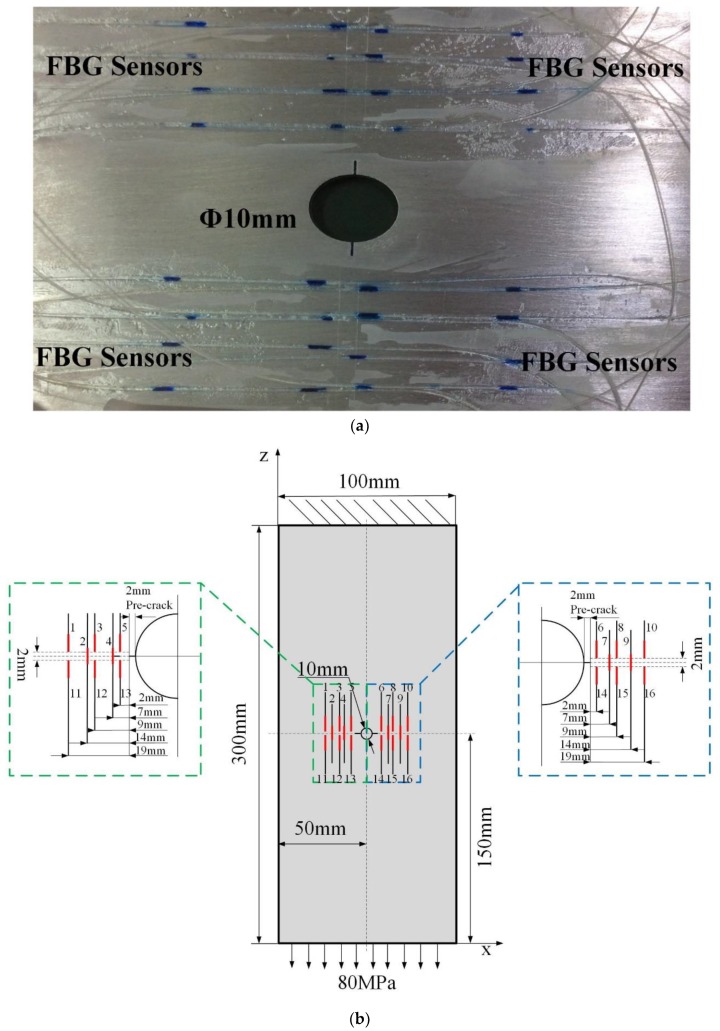

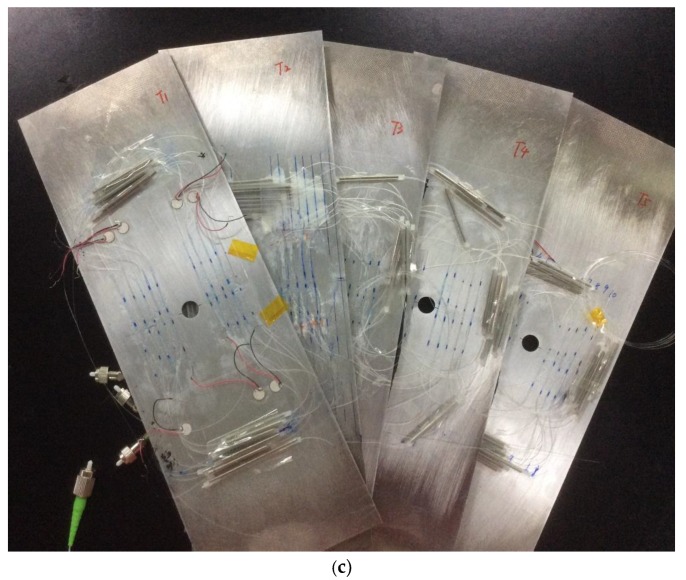
(**a**) Schematic of the aluminium specimen. (**b**) The FBG sensor network design. (**c**) The parallel specimens.

Five parallel experiments with the same design and operation were carried through to reduce the uncertainty (shown in Figure 7c). The coupons (shown in Figure 7a,b) are made of aerial 2024-T3 aluminum alloy with dimensions of 300 × 100 × 2 mm, and were manufactured at the Beijing Institute of Aeronautical Materials. A 10 mm in diameter hole was drilled in the intermediate plate, and a 3 mm electric discharge machining (EDM) pre-crack was produced on both sides of the hole to accelerate the expansion of fatigue cracks. The yield strength of the specimen materials is 360 MPa, the ultimate strength is 490 MPa, the Poisson’s ratio is 0.33 and the Young’s modulus is 72 GPa. According to a previous study [44], pre-cracks around holes are highly relevant to the stress concentration and will be a hot spot to develop fatigue damage.

### 3.2. FBG Sensors Network Design

The FBG sensor network design is critical for damage detection. Considering the effect of the crack damage on the axial strain profile, the FBG sensors are placed at the end of the crack tip in this paper. Based on the previous finite element analysis under different crack lengths [8] showing that when some FBG sensors were arranged uniformly along the crack propagation direction with a distance of 1 mm, the grating can sense the crack-tip strain singularities with sensitivity, the other FBG sensors were symmetrically distributed along the radial direction that the crack would propagate along. Thus, sixteen FBG sensors were uniformly symmetrically bonded by a liquid cyanoacrylate adhesive on each side of the hole. The detailed sensor layout is shown in Figure 7b, where the red lines represent FBG sensors near the target region. The Young’s modulus of the FBG sensor adhesive is 0.0017 GPa, and the length is 10.01 mm. 

### 3.3. Experimental Setup

**Figure 8:**
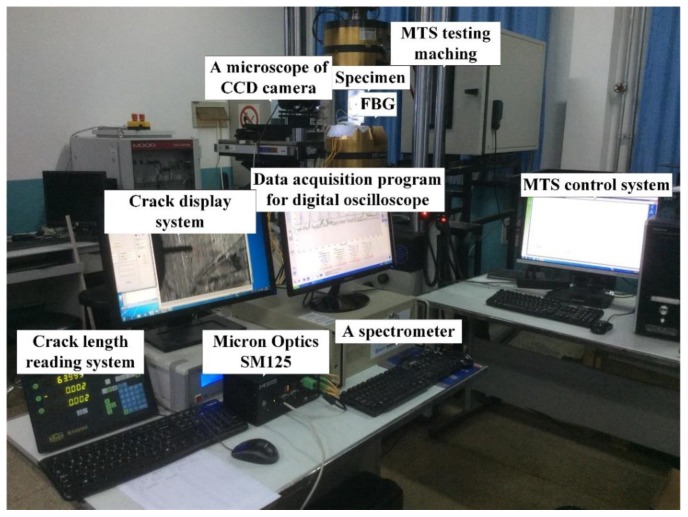
Experimental setup for the FBG sensor damage detection system.

The experimental platform for fatigue hole-edge crack damage detection contains three major parts: an optical sensing and data acquisition system, a fatigue crack measurement system, and a fatigue load-cycling system, which are shown in Figure 8. 

FBG sensors (FSSR5025) from the Changcheng Institute of Metrology and Measurement (Beijing, China) were used to acquire the strain information in the regions vulnerable to fatigue crack damage. The optical demodulator system (SM125, Micro Optics Inc., Danbury, CT, USA) was used to record the reflection intensity spectrum at various crack lengths from 1–30 mm; the measurement accuracy is 1 pm resolution and the stability is less than 1 pm. A spectrometer of 1 pm resolution is used to obtain the real FBG reflection spectrum. The real cracks were monitored by a traveling optical microscope with a charge coupled device (CCD) camera during the fatigue cycling load testing by a hydraulic MTS fatigue machine at constant temperature. The theoretical peak value is equal to the standard spectrometer results. A constant amplitude tensile loading was applied to the bottom of the specimens with the top boundary fixed, the maximum amplitude was 75 MPa, the stress ratio was 0.1 and the load frequency was 5 Hz. In addition, the fatigue testing experiments were paused under the maximum strain for data acquisition, and the processing was repeated twice to eliminate any operational error during each of the pauses and account for system hysteresis. 

Fiber Bragg gratings are some of the most used devices to measure strain and deformations in many smart structures. In these applications, the FBG measures the total deformation including strain due to forces applied to the structures as well as thermal expansion [45]. The relative Bragg wavelength shifts in response to axial strain change ∆ε and temperature change ∆T are defined as $∆\lambda_{\varepsilon }/\lambda_{B}$ and $∆\lambda_{T}/\lambda_{B}$, respectively, which can be described as the following formula:

(6) $$\frac{∆\lambda_{\varepsilon }}{\lambda_{B}}=\left(1-P_{\varepsilon }\right)∆\varepsilon$$

(7) $$\frac{∆\lambda_{T}}{\lambda_{B}}=\left(\alpha_{f}+\delta_{f}\right)∆T$$

where $P_{\varepsilon }=0.22$ is the effective photoelastic coefficient of the fiber glass; $\alpha_{f}$ and $\delta_{f}$ are the thermal-expansion coefficient and the thermal-optic coefficient of common single-mode fiber, respectively.

In practice, The Bragg wavelength of an FBG sensor depends mainly on strain, but it shifts slightly with any temperature change. With a change of 1 °C, the measurement strain typically has an error of 11 με [46]. On the other hand, this cross-sensitivity of the FBG with temperature may become a problem in applications that require the discrimination the total deformation. For accurate strain measurementa by FBG sensors, it is necessary to compensate the influence of temperature change. In order to overcome this inconvenience, researchers proposed many schemes by mixed fiber gratings [47], different-dimeter fiber grating [48], and fiber gratings partly embedded in glass tubes [49,50]. 

In this paper, the damage monitoring experiment aims to detect the strain effect caused by crack propagation with FBG sensors. Therefore, the thermal-strain cross effect may be limited in the experiment design part and the measurement signal needs to be processed to eliminate the temperature influence.

In order to control temperature effects, the damage monitoring experiment was carried out under the constant temperature with a central air-conditioning control system set at 27.6 °C, and data acquisition time within seconds, therefore the slight temperature fluctuations during the process are assumed negligible. 

Additionally, the reference signals of each FBG which was bonded on the specimen were acquired before the fatigue experiment under no crack environment with the loading fixed at 75 MPa, and the temperature set at 27.6 °C. Then, the crack propagation after fatigue cycles and the damage signals of each FBGs were detected at different crack lengths under fatigue testing paused at 75 MPa with same temperature and bending state of the reference signal. Besides, the central wavelength shift of each FBG is defined as the difference between the damage signals and the reference signals. Then the central wavelength shift at every crack of the FBGs are subject to the strain change without thermal expansion and bending effect.

**Figure 9:**
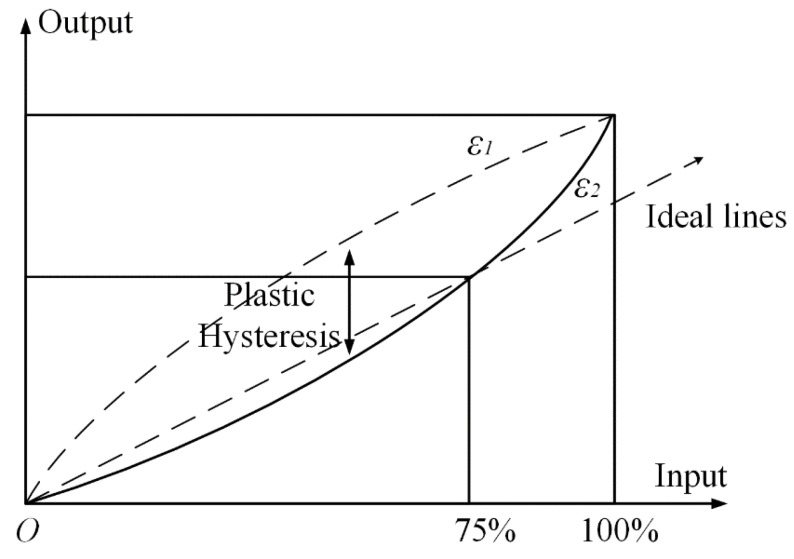
Schematic diagram of hysteresis effect.

Furthermore, the Bragg wavelength relative shift is proportional to the axial strain, or in other words, FBG converts strain into wavelength shifts, which is an absolute parameter, immune to optical power drifts along the measurement chain. In addition, strain is exerted on the attached optical sensor, which in turn modifies the sensor spectral response. However, on account of the hysteresis error of the optical measuring system of this sensor due to the hysteresis and non-linear phenomena taking place in such substrate materials do not obey Hooke’s law perfectly [51]. In details, when substrate materials are under an external force, the micro-strain between every micro-grain inevitably displays inhomogeneity [52]. Additionally, strain lagging the stress within the elastic deformation range may appear. Moreover, there is a non-strict linear relationship between strain and stress and the strain curve in loading process $\varepsilon_{1}$ does not coincide with that in unloading process $\varepsilon_{2}$, which is shown Figure 9. Moreover, the plastic hysteresis can be defined as the difference between $\varepsilon_{1}$ and $\varepsilon_{2}$ [53].

Even the hysteresis is a limiting factor concerning the global performance of the device, the accurate and reliable modelling of its effects allows the sensor to achieve sufficient accuracy. To this end, plastic materials are accurately modelled in order to compensate for hysteresis. However, recently, hysteresis modelling has seen great progress [54,55], resulting in suitable techniques to model hysteresis compensation that can be employed, such as the compensation block [56] and a proposed parabolic approximation technique [57], which allows the device to show a full linear response. 

In this paper, the hysteresis effect limiting method is applied in the developed damage measurement experiments and yielded to a more linear response of the sensor for a reliable reconstruction of the strain field. Firstly, the data acquisition under the maximum strain stabilization paused with the loading fixed as 75 MPa was proposed and the processing was repeated twice to eliminate the operational errors during each of the pauses. This process limited the system hysteresis under the loading and unloading cycles to be minimum. 

In addition, the relation between the component strain and the strain on a surface-attached optical fiber is governed by the effectiveness of shear transfer through the adhesive and the polymeric coating on the optical fiber [58]. When considering adhesive affect strain transfer in the surface-attached sensor under the loading state and the sensor’s performance may be reduced due to single-input-multiple-output. To decrease the adhesive effect, FBG sensors were bonded by a liquid cyanoacrylate adhesive on the specimen due to the fact the Young’s modulus of the adhesive that is between the specimen and sensors is close to the Young’s modulus of the specimen material (the Youn’s moduli of the adhesive and FBG coating are 72 GPa, 0.00175 GPa and 0.0017 GPa), therefore, the strain can be perfectly transferred between the plate and FBG sensor.

## 4. Results and Discussion

The damage monitoring experimental procedure was carried out as described in Section 3, and in this section, the healthy and damage signals received are used to evaluate the effectivity of the central wavelength algorithm and monitor the damage crack propagation.

### 4.1. Evaluate the Effectivity of the Central Wavelength Algorithm

To validate the feasibility and dependability of the multi-peak central wavelength detection algorithm, the traditional algorithms such as direct-peak located algorithm, centroid algorithm, polynomial fitting algorithm and Gaussian fitting algorithm are applied for comparison. The traditional algorithms have high requirements for the spectral type and the environmental noise. In order to evaluate the algorithm effectively, the detected error and the standard deviation are proposed as the assessment indicators. The detected error is the discrepancy between the measured peak value and the theoretical peak value equaling to the standard spectrometer results. The detected error [59] occurs because the measurement of the data is not precise due to the algorithm. In the mathematical field of numerical analysis, the absolute value [60] and the relative value [61] of the measured and theoretical peak values were expressed as the algorithm error, which is described as follows:

Absolute error = measured peak value-theoretical peak value(8)

(9) Relative error=|measured peak value−theoretical peak value|/theoretical peak value

Considering the stability of the optical demodulator system (SM125) is 1 pm., the accuracy of the signals acquired by the system would be influenced, then the highest precision of the peak seeking algorithm would also be limited to pm level. Moreover, in order to evaluate the peek seeking algorithms’ performance, the data processed by the different algorithms were the same. Therefore, the uncertainty fluctuation of the data has the same variation effect on each of these algorithms. In summary, the absolute error and relative error can be presented as the assessment indicators. 

**Figure 10:**
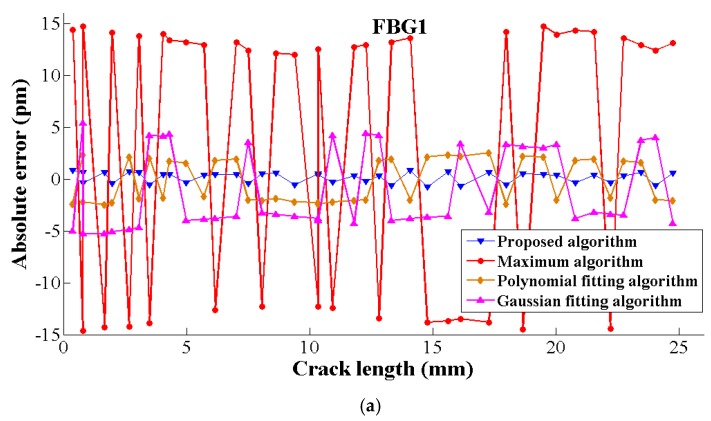

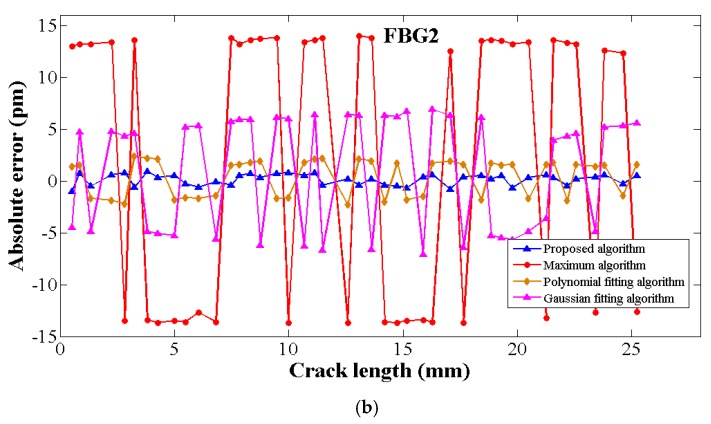
The absolute error of five peak seeking algorithms at different crack lengths. (**a**) The absolute error of five peak seeking algorithms in FBG1, (**b**) The absolute error of five peak seeking algorithms in FBG2.

It is observed that the proposed peak seeking algorithm have the best performance in algorithm precision with the minimum absolute error within the range of (−2 pm, 2 pm), while the maximum algorithm had a maximum error in the range of (−15 pm,15 pm), as shown in Figure 10. Furthermore, the absolute error contains positive and negative errors considering the power fluctuation or other signal measurement effects. 

**Figure 11:**
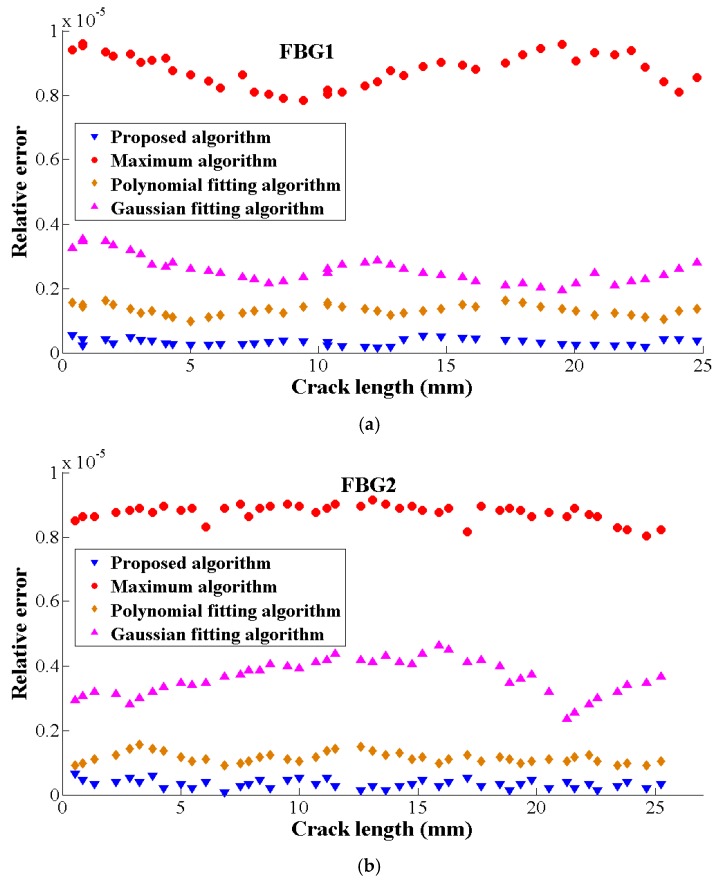
The relative error of five peak seeking algorithms at different crack lengths. (**a**) The relative of five peak seeking algorithms in FBG1, (**b**) The relative of five peak seeking algorithms in FBG2.

Additionally, considering the accuracy of the measurement equipment, the relative error results shows that the proposed algorithm has the minimum value while the maximum algorithm has the maximum relative error (in Figure 11). Therefore, the accuracy and the stability of the proposed algorithm shows superiority over other algorithms. Even though they have the same computational complexity when considering the vector input size, the proposed algorithm performs better due to its implementation details.

When algorithm robust is considered, the detection wavelength standard deviation σ given by Equation (10) is proposed as an indicator:

(10) $$\sigma =\sqrt{\frac{1}{N}\overset{N}{\underset{i=1}{\sum }}{\left(\lambda_{a}-\lambda_{b}\right)}^{2}}$$

where σ is the standard deviation of the calculated peak wavelength and the theoretical peak wavelength, *N* is the number of the spectrum collection times, $\lambda_{a}$ is the peak wavelength calculated by algorithm and $\lambda_{b}$ is the theoretical peak wavelength. 

**Figure 12:**
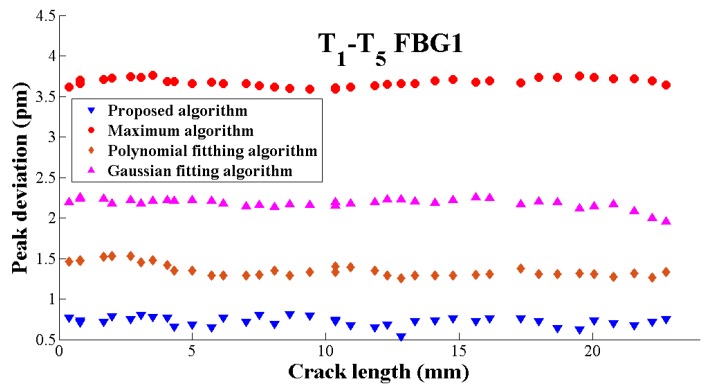
The standard deviation of five peak seeking algorithms at different crack lengths.

It is observed that the proposed algorithm has the smallest σ, which shows the best robustness of five overall algorithms in Figure 12. The reasons for the other four algorithms poor performance are various that the direct-peak located algorithm precision may be affected by the noise, additionally, the fitting algorithm such as the Gaussian fitting algorithm and the polynomial fitting algorithm do not take the FBG asymmetry spectrum into consideration. 

The analysis of the experiment results shows that the proposed algorithm guarantees the best combination of versatility, precision and accuracy. Consequently, the algorithm in this paper could satisfy the requirements of detecting the promising central wavelength. 

### 4.2. Analysis the Central Wavelength Shifts with Crack Propagation

The central wavelength shifting of the FBG reflection spectrum is chosen as the damage feature due to its sensitivity characteristic to the strain distribution caused by crack propagation. 

The wavelength shift, $∆\lambda_{B}$, is a parameter related to the strain level in the structure, but it is dependent on the loading and geometry configuration. However, the rapid increase in the magnitude of $∆\lambda_{B}$ is caused by damage event that reduces the stiffness of the structure. Based on the coupled mode theory, the central wavelength determined by the FBG period Λ and the effective refractive index $n_{eff}$, At constant temperature, the load-induced Bragg wavelength $\lambda_{B0}$ shifts with the grating period Λ for the light propagating direction and the mean effective index of refraction, $n_{o}$:

(11) $$\lambda_{B0}=2n_{o}\Lambda$$

**Figure 13:**
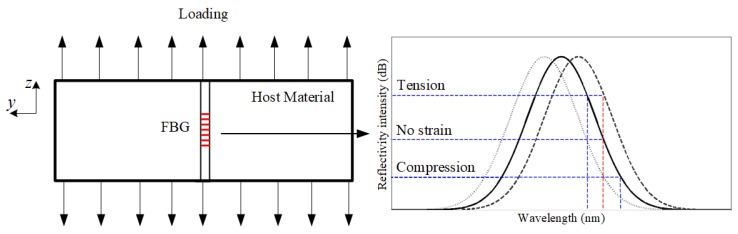
The force analysis of the FBG sensor when axial places on the aluminium plate under axial loading and the FBG reflection spectrum shift under no strain field, tension stress field and compression field.

What is more, according to the previous research [8], no crack is present and a uniform strain, $\varepsilon_{z}$ builds up around the grating area as the structure is loaded. The FBG response is a uniform wavelength shift in the reflected peak, ∆λ. Moreover, there is a linear relationship between the change of the central wavelength and the axial uniform strain $\varepsilon_{z}$ when the grating axial placed far away the crack tip (shown in Figure 12). The relation is expressed as Equation (12):

(12) $$\frac{∆\lambda_{B}}{\lambda_{B0}}=\left(1-\frac{n_{0}^{2}}{2}\left(p_{12}-\nu \left(p_{11}-p_{12}\right)\right)\right)\varepsilon_{z}=\left(1-p_{e}\right)\varepsilon_{z}=K_{\varepsilon }*\varepsilon_{z}$$

where the new strain optic constant $p_{e}$ is determined by experimentally. This relation points out that the sift in wavelength of the Bragg peak is proportional to the applied axial strain. When it is uniform, shifts occur without modification of the initial spectrum shape (shown in Figure 13).

When the crack propagation to the FBGs, the materials around the crack tip under high stresses field, and the effect of the stress on z direction shrinkage is constrained by the surrounding materials. Then the strain filed at the crack tip $\varepsilon_{z}$ is small, represented to as a plane-strain state, leading to the characteristic singular stress distribution at the crack tip.

**Figure 14:**
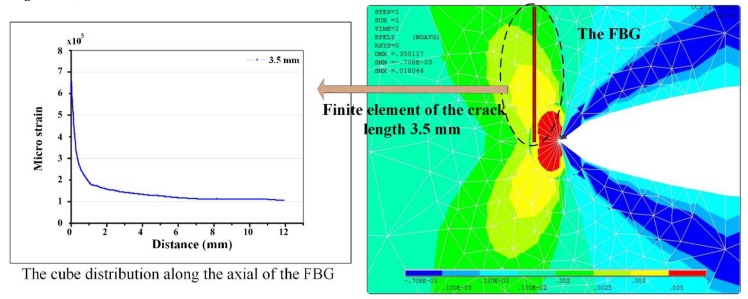
The stress distribution at the crack tip extracted by the Abaqus software.

**Figure 15:**
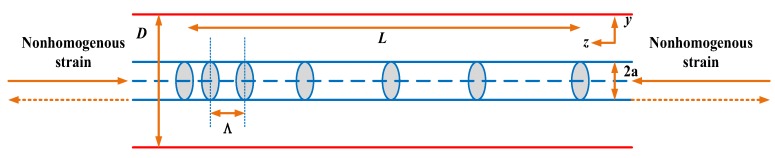
Axial nonuniform stress structure diagram of the FBG.

According to the finite element analysis of fatigue crack propagation near the FBG sensor by ANSYS software, the fiber grating senses the nonhomogeneous strain (shown in Figure 14), the reflectivity among the grating and the pitch distance has been non-uniformity changed (shown in Figure 15). 

On constant temperature condition, when the nonhomogeneous strain distribution along the grating, the central wavelength changes no more than a linear function with the strain. In a non-uniform strain field, where the strain components are functions of the axial position z. Consequently, the Bragg wavelength shifts have a function of strain in z direction, then the specific expression is Equation (13):

(13) $$\frac{∆\lambda_{B}\left(z\right)}{\lambda_{B0}}=\frac{\lambda_{B}\left(z\right)-\lambda_{B0}}{\lambda_{B0}}=\varepsilon_{z}\left(z\right)-\frac{n_{0}^{2}}{2}\left(p_{12}\varepsilon_{z}\left(z\right)+\frac{1}{2}\left(p_{11}+p_{12})(\varepsilon_{x}\left(z\right)+\varepsilon_{y}\left(z\right)\right)\right)$$

While the spectrum response of a Bragg grating is quite simple to interpret when the strain is homogeneous, its response in the presence of strain gradients becomes more complicated to characterize. Indeed, the reflection spectrum becomes broadened with multiple peaks and is said to be chirped.

**Figure 16:**
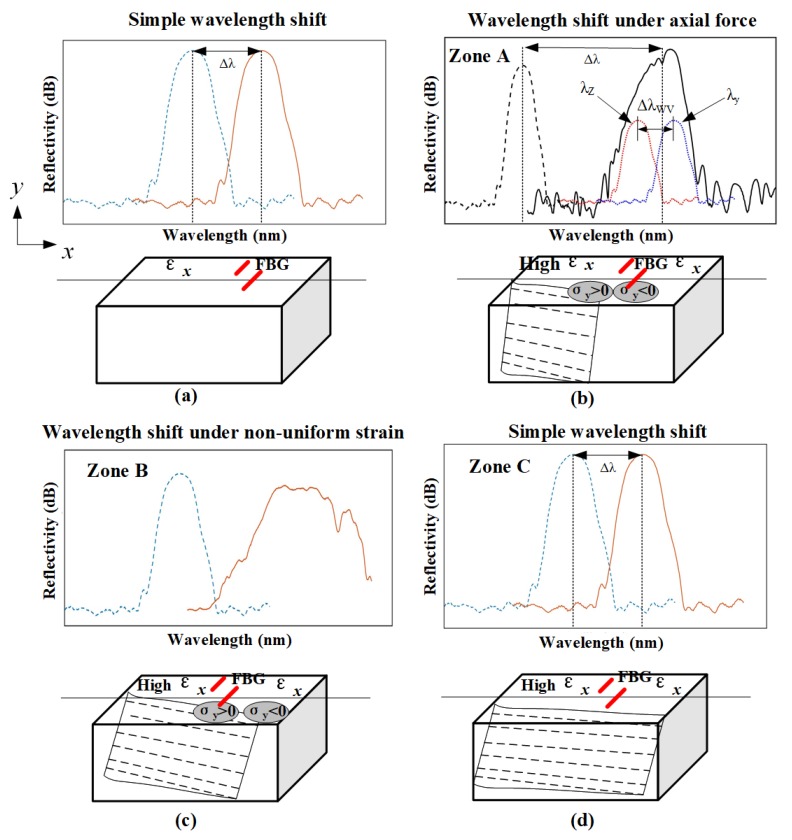
Schematic of the stress field under the crack propagation before cross and after cross the FBG sensors. (**a**) The simple wavelength shift, (**b**) The wavelength shift under axial force, (**c**) The wavelength shift under none-uniform strain, (**d**) The simple wavelength shift.

The crack length versus the FBG reflection spectrum is presented in Figure 16. This is worth noting that in the initial stage of crack propagation, considering the structure loading or crack singularities field distance from the grating is enough, the reflection spectrum displays a very narrow symmetric Gaussian shape, and only the central wavelength makes a shifting with the crack (in Figure 16). Then, when the crack propagates close to the FBG, in the plastic zone located at the head of the crack tip, the grating senses a higher average strain and the central wavelength shifts to a higher wavelength. Additionally, when the grating is locates in the crack singularity field, the inhomogeneous strain field in the grating direction will change, the strain gradient load on the grating increases, then the central wavelength will move in a long wavelength direction. The original grating was broken when it reached the maximum chirp status and the subordinate peak initially appeared in the short-wavelength direction with the bandwidth broadened (in Figure 16 zone A) [8]. If the crack continues to grow, the grating will gradually experience the influence of the crack singularity (the region dominated by stress concentration). Non-linear behaviours of the structure which are caused by the material nonlinearity (plasticity) and a change of configuration can be sensed by the grating gauge when the crack crosses it. The tensile stress loaded on the grating and the central wavelength continues shifting to the longer wavelength as the peak splitting in the FBG reflection spectrum increases, then subordinate peaks appear in the long-wavelength direction (in Figure 16 zone B). Moreover, it is notable that the different FBGs layouts present different reflection spectrum results when the crack shifts away from the sensors (in Figure 16 zone C).

**Figure 17:**
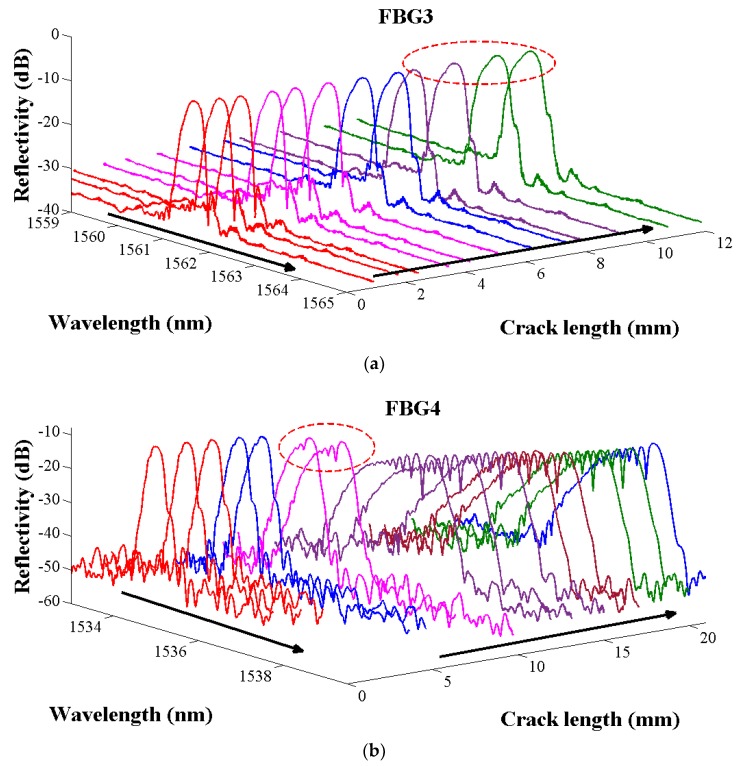
The FBG3 and FBG4 reflection spectrum in various crack lengths. (**a**) The reflection spectrums of FBG3 in different crack lengths, (**b**) The reflection spectrums of FBG4 in different crack lengths.

It is possible to observe some differences in the evolution of the wavelength shift because the position of the sensor and the crack related to the applied moments is different. The FBGs are placed perpendicular to the crack propagation direction with a distance of 1 mm, and their reflection spectrum gradually recovers its original shape, then the spectrum returns to the Gaussian shape and the central wavelength tendency shift to the short-wavelength direction (in Figure 17a, the FBG spectrum in red cycle are presented the moments that crack propagates to the FBG3). 

The FBGs are bonded at the place where the crack crosses, and it is obvious that the reflection spectra continue to deform when the crack passed through fiber grating owing to the damaged gauges (in Figure 17b, the FBG spectrum in red cycle are presented the moments that crack propagates to the FBG4). However, the wavelength shift continues to vary, following the increase of load and strain in the specimen. 

**Figure 18:**
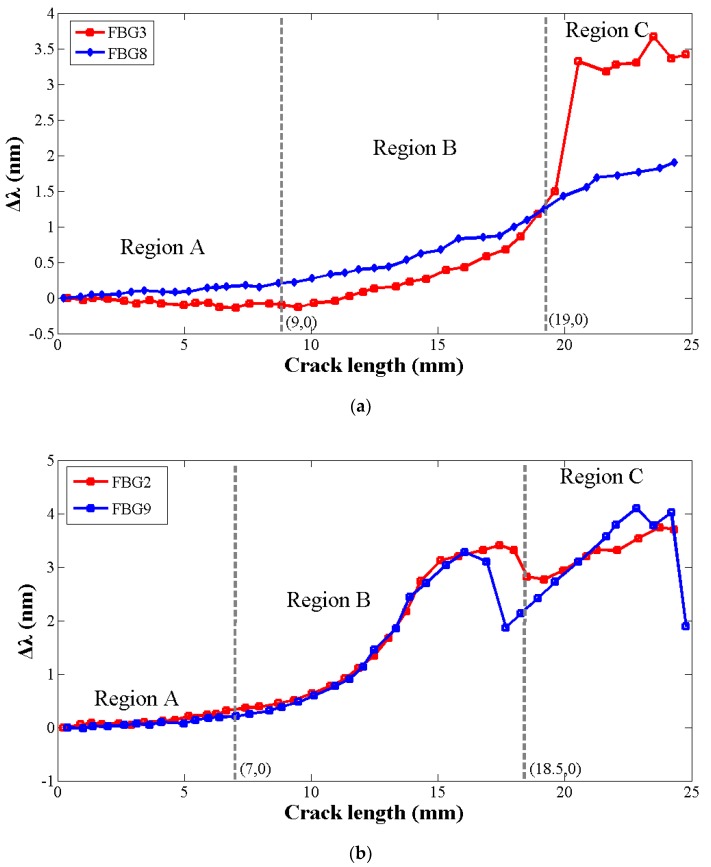

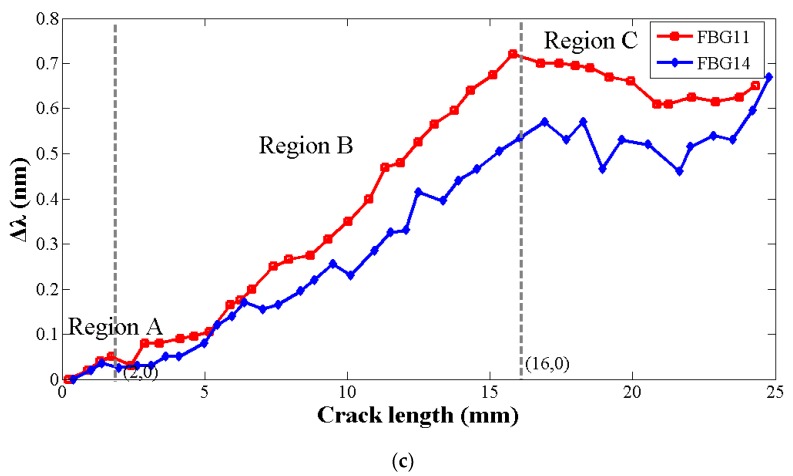
The central wavelength shifting of different FBG in various crack lengths. (**a**) The central wavelength shifting of FBG3 and FBG8, (**b**) The central wavelength shifting of FBG2 and FBG9, (**c**) The central wavelength shifting of FBG11 and FBG14.

It is observed that the central wavelength shifting of the FBG sensors located symmetrically along both sides of the hole show similar turning points and increasing tendency with the crack (in Figure 18). In the initial stages of crack propagation, the shift of the central Bragg wavelength was less than 0.1 nm during this process as a result of a small offset between the center of the FBG and the intersection point between the FBG and the neutral line. Then, with the crack propagation, the central wavelength increases as the average strain sensed by the grating gradually increases (in Figure 18, region A). Additionally, when the crack extends close to the grating and the gauges sense increasing non-homogeneous strain, the curve rises quickly (in Figure 18, region B). Therefore, it is obvious that a jump in the wavelength shift is observed when the crack passes the position of the gratings. The crack changes the local compliance of the material and load distribution, making the area that surrounds the sensor less stiff and more deformed, therefore, an increase in the strain is measured. 

In the end, for the FBGs bonded up and down perpendicular to the crack direction, the change of central wavelength becomes steady and a back return trend is seen when the crack passes away from the sensor, as the field around the grating returns to an approximately uniform strain condition, shown in region C in Figure 18a,c. For the FBGs crossed by the crack, such as FBG2,9, the spectrum maintains its deformation shape and the central wavelength still increases, as shown in region C in Figure 18b.

The processing period is divided into three stages: gradual increase, quick increase and steady increase. Since the turning points are different in the three periods of each curve on account of the sensor placed at different positions, the analysis of central wavelength slopes and turning points in the three stages caused by the changing average strain and strong stress concentration during the crack extension could help us to judge whether the crack is approaching the FBG sensors’ position. However, when the sensors are in the symmetrical position on both sides of the hole, the two curves are also imperfectly superposed due to the signal uncertainty. The reasons for the differences are many, such as the discrepancy of elastic properties, the crack orientation, as well as the geometry features, and an analysis of these factors will be studied in the future. In this way, the central wavelength can be used to monitor whether the crack is propagating towards the FBG sensors. Accordingly, the central wavelength can be regarded as a damage parameter to monitor the fatigue crack propagation lengths when the FBGs are located at the near-end stress field.

## 5. Conclusions

In this work, a self-adaptive multi-peak detection algorithm is developed to extract the damage characteristic variation of the central wavelength to detect the crack damage status in aluminum alloy plates using FBG sensors. The peak searching results reflect that the proposed algorithm presents a significant improvement in accuracy and robustness compared to other conventional methods. Additionally, the ability of fiber Bragg gratings bonded on aluminum materials to detect and track cracks by connecting the response of a sensor to a specific fracture/damage phenomenon was demonstrated. The central wavelength shifts detected from the FBG reflectivity spectrum are sensitive to the uniform and non-uniform strain distribution along their longitudinal direction, and can be used as a tool to monitor and evaluate different damage status during a crack growth event in quasi-static tensile tests for aluminum plates. The curve of the characteristic parameter variation during a crack growth event is divided into three regions: gradual increase, quick increase, and steady increase, corresponding to the initial stages of crack propagation at a far distance from the FBGs, the crack proportion near and through the FBGs stage, and the cracks far away from the FBGs stage, caused by the tensile and compressive stress in the crack tip plasticity area. This technique was successfully validated under experimental cycle loading conditions, and very promising results demonstrate the possibility of crack quantitative detection based on FBG sensors.
